# The Impact of Avoidant/Disengagement Coping and Social Support on the Mental Health of Adolescent Victims of Sexual Violence in Eastern Congo

**DOI:** 10.3389/fpsyt.2020.00382

**Published:** 2020-06-19

**Authors:** An Verelst, Sarah Bal, Maarten De Schryver, Nanc Say Kana, Eric Broekaert, Ilse Derluyn

**Affiliations:** ^1^Centre for Children in Vulnerable Situations, Department of Social Work and Social Pedagogy, Ghent University, Gent, Belgium; ^2^Ghent University Hospital, Ghent University, Gent, Belgium; ^3^Faculty Research Support Office, Ghent University, Gent, Belgium; ^4^Department of Special Needs Education, Ghent University, Gent, Belgium

**Keywords:** sexual violence, mental health, adolescent girls, coping, family support, social support

## Abstract

**Introduction:**

Eastern Congo has been affected by armed conflict for decades while the rampant use of sexual violence has left many women and girls dealing with a wide range of consequences of sexual violence. For adolescent victims the psychosocial impact of sexual violence is devastating. However, the role of avoidant/disengagement coping and family support on the mental health impact of sexual violence remains unclear.

**Methods:**

The study design was a cross-sectional, population-based survey in which 1,305 school-going adolescent girls aged 11 to 23 participated. Mental health symptoms (IES-R and HSCL-37A), family support (MSPSS), avoidant/disengagement (Kidcope), war-related traumatic events (ACEES), experiences of sexual violence, daily stressors, and stigmatization (ACEDSS) were administered through self-report measures. Hierarchical multiple regression analysis was carried out with mental health outcomes as dependent variables for different types of sexual violence. Finally, several ANCOVA models were defined to explore possible interaction effects of avoidant/disengagement coping and family support with stigmatization, daily stressors and war-related traumatic exposure.

**Results:**

For girls who did not report sexual violence, *avoidant/disengagement coping* has a direct negative effect on all psychological symptoms. For victims of sexual violence, when high levels of stigma were reported, avoidant/disengagement coping possibly served as a protective factor, as shown by the interaction effect between avoidance/disengagement coping and stigmatization on mental health outcomes. In victims of sexual violence however, high levels of daily stressors combined with avoidant/disengagement strategies showed a strong increase in posttraumatic stress symptoms. Interestingly, the mental health impact of sexual violence was not mitigated by support by family members. For girls who reported a nonconsensual sexual experience without labelling it as rape and at the same time testified to have a lot of family support, there was a positive association between stressors (daily stressors, stigma, and war-related trauma) and posttraumatic stress symptoms.

**Conclusions:**

These results of this study underwrite to the importance of looking beyond the straightforward negative impact of avoidant/disengagement coping strategies on mental health in adolescent victims of sexual violence. While avoidant/disengagement coping can have a negative impact on psychosocial well-being on adolescent victims of sexual violence, in case of high levels of stigmatization it can as well protect them from posttraumatic stress or anxiety. Furthermore these findings speak to the importance of exploring the diversified relationship between risk and protective factors, such as avoidant/disengagement coping strategies and family support, that shape the mental health impact of sexual violence in adolescent victims.

## Introduction

A decade long conflict has deeply harmed and devastated the Congolese society; its population afflicted by multiple human rights abuses (1, 2). The use of strategic violence against civilians in this warring context has affected families, kinship, and community bonds, thereby pervasively disrupting social ties (3). One of these “weapons of war” is sexual violence, extensively—up until today—used as a war tactic by many armed groups in eastern Congo (4, 5). Moreover, a “normalization” of rape has also been noticed, with a substantial increase of reported sexual violence by civilian perpetrators (6, 7). However, different forms of sexual violence by civilian perpetrators are often silenced, due to socio-cultural norms and the prevailing discourse framing sexual violence as a weapon of war (7). This could also influence how victims label an experience of sexual violence. Even when women undergo sexual experiences that are legally considered as rape, many of them will not label it as such (8–12). This labeling of an unwanted sexual experience has important implications for victims' mental health, although findings about the direction of this impact are inconsistent (11, 13–16). Nevertheless, overall, experiences of sexual violence have highly detrimental effects for victims' mental well-being (11, 17–20), irrelevant of how it is labeled. Victims of sexual violence in war-torn eastern Congo has shown that both girls who label a nonconsensual sexual experience (NCSE) as rape as well as those who do not label their sexual violence experiences as “rape,” report very high levels of psychological symptoms (21).

The large variation in mental health consequences of sexual violence (22) has led many scholars to investigate factors that might impact mental health. Risk factors of other war-related traumatic events, daily stressors, and (widely reported) negative social reactions have been identified as contributing to mental health outcomes of sexual violence in war settings (20, 22, 23). Recent studies point in particular to how the various ways victims of sexual violence are stigmatized, rejected, and excluded has a detrimental effect on their mental health (20, 21, 24–26).

However, there is a scarcity of literature investigating the impact of possible protective factors, such as coping and social support, for adolescent victims of sexual violence in (post)conflict contexts.

Coping mechanisms are the cognitive and behavioral strategies applied when faced with stressful events (27). Coping strategies have been described in different ways, but are generally positioned into two dimensions, namely, approach/engagement strategies (cognitive or emotional activity toward a stressful event or object or the emotional or cognitive reaction a person has to it; e.g., problem solving, cognitive restructuring) versus avoidance/disengagement strategies (cognitive or emotional activity away from a stressful event or objector the emotional or cognitive reaction a person has to it; e.g., distraction, social withdrawal) (28). Approach strategies have been associated with fewer psychological symptoms and a smoother recovery after sexual violence (29, 30). In general, while avoidance strategies may be considered adaptive to reduce the stress directly after the traumatic event, in the long term they could lead to more mental health problems (29, 31–33). This is also applicable in the case of victims of sexual violence (33–36). Research with adolescent victims of sexual violence has illuminated that victims of sexual violence have a tendency to use avoidance strategies (37), and that this is even more so for victims who label their NCSE as rape in comparison to victims who do not label it as such (11). While studies in nonwar affected areas have strongly supported these findings, namely, that avoidance coping is associated with more psychological problems, some studies, especially in war-torn areas, have generated evidence that avoidance can lead to fewer psychological problems such as depression and anxiety (31, 38). A study of Mels and colleagues (39) assessing the impact of coping on mental health issues in eastern Congolese adolescents found that avoidance/disengagement coping was associated with a reduction in symptoms of posttraumatic stress and anxiety in older adolescents. However, the particular role of avoidant/disengagement coping in the mental health of adolescent victims of sexual violence has not yet been investigated in (post) war contexts.

Social support—''the availability of components of support from interpersonal relationships'' [(40):1273]— has generally shown to be a protective factor for mental health outcomes of sexual violence (41, 42). Also, family support in particular can be a protective factor to the mental health consequences of rape in adolescent victims (43). Additionally, victims who label their experiences of sexual violence as rape have been shown to look for social support more often (11). However, in (post)conflict contexts, such as eastern Congo where rape is used as a weapon of war to rupture social ties, the disturbed family and community support structures are often still too weak to provide victims of sexual violence with the support they so greatly need (23). Being raped decreases a girl's marriageability, and thus risks having economic and social consequences for the family (44). While family support can have a protective effect on victims' mental health after sexual violence, the adverse impact of negative social reactions on victims' mental well-being might be even stronger (40, 45–47).

This study, therefore, strives to increase the understanding of the use of avoidant coping and family support as well as the impact on the mental health of adolescent victims of sexual violence living in the distinct setting of war-affected eastern Congo. Hereby also taking into consideration their association with the following risk factors: daily stressors, war-related traumatic exposure, and stigmatization. From these findings, implications for interventions will be drawn.

## Methods

### Participants and Procedure

The study was conducted in the current province of Ituri, in Eastern DRC, a region where armed conflicts have caused havoc for the last decades (1, 48). While acknowledging that also boys face considerable levels of sexual violence in this region (49), this study focuses on adolescent girls. This choice was made in close collaboration with the local expert team guiding this study. A boy's responses could be highly influenced by taboos regarding sexual violation of boys, rendering this method (self-report measures in a classroom setting) less applicable for boys. Across the large region of Ituri's main city, Bunia, 22 secondary schools in all 10 neighborhoods, were selected using stratified sampling in relation to location (rural, suburban and urban regions). All of the selected schools agreed to participate. Per school all the female pupils from the second and third year of high school were invited, informed and consented to take part in the study (n = 1,304). Of the participants, aged 11 to 23, with a mean age of 15.89 (SD = 1.54), 14.0% (n = 183) of the sample confirmed being raped, while 24.2% (n = 315) of the sample mentioned having experienced a NCSE which they did not label as rape (**Table 1**). Some socio-demographic differences were found between the three groups (**Table 1**).

**Table 1:** Socio-demographic characteristics and stressful experiences of the participants.

	Total group (n=1,304)	No sexual violence (n=806)	Rape (n=183)	Nonconsensual sex (n=315)	*F/χ^2^*
Age^†^	15.89 (1.54)	15.73 (1.49)	16.34 (1.51)	16.04 (1.63)	13.90**
Socio-economic status					21.08**
Brick house	600 (46.4%)	404 (50.5%)	86 (47.5%)	110 (35.3%)	
Nonbrick house	693 (53.6%)	396 (49.5%)	95 (52.5%)	202 (64.7%)	
Parental availability					20.20**
Both parents alive	781 (78.93)	486 (80.7%)	103 (67.8%)	192 (79.0%)	
One or both parents deceased	216 (21.7%)	116 (19.3%)	49 (32.2%)	51 (21.0%)	
War-related traumatic exposure (ACEES)^†^	2.83 (2.43)	2.19 (1.90)	4.71 (3.05)	3.16 (2.45)	86.62
Daily Stressors (ACEDSS)^†^	5.34 (3.31)	4.57 (2.98)	7.78 (2.98)	6.08 (3.11)	201.12*
Stigmatization (ACEDSS)^†^	3.95 (3.45)	2.82 (2.50)	7.53 (4.28)	4.90 (3.37)	200.33

N(%); ^†^Mean (SD); *p < .01, **p < .001; Rape, participant who reported experiences of rape; Nonconsensual sex, participants who reported nonconsensual sexual experiences, but did not label these as “rape”; ACEES, Adolescent Complex Emergency Exposure Scale; ACEDSS, Adolescent Complex Emergency Daily Stressors Scale.

The questionnaires were administered during a 60–90 min class period, while the boys of the respective classes were engaged in other activities organized by the teacher. A description of the study was provided to the participants followed by obtaining written informed consent. During the completion of the self-report questionnaires, the researcher and a research assistant or two research assistants were present. Questionnaires were administered in French, since this is the official language in secondary schools, and a pilot study showed that the students preferred French questionnaires over the translated Kiswahili versions. Questionnaires were self-administered while thoroughly guided and structured by the research assistants. To promote interresearcher reliability 90 h of extensive theoretical and practical training was provided to all research assistants. The researcher provided her contact details to participants, as well as information on local psychological support projects for those in need of further professional care. The researcher had a large network of professional psychosocial professional services that were used to refer participants of this particular study to. Ethical approval for the study was given by the Ethical Committee of the Faculty of Psychology and Educational Sciences, Ghent University.

### Measures

Six self-report questionnaires, all culturally adapted and some constructed for use in eastern Congo (50) were administered. First, a socio-demographic questionnaire investigated variables such as age, housing situation (as an indicator of participants' socio-economic status), and parental availability.

Second, the Adolescent Complex Emergency Exposure Scale (ACEES) was used, as it was developed to measure exposure to potentially traumatic war-related events in eastern Congolese adolescents (50). The ACEES measured exposure to 14 context-specific potentially traumatic war-related events (yes/no), such as having witnessed people being killed, being separated from family and having witnessed rape. In addition to this questionnaire, specific questions regarding experiences of sexual violence were added. Besides the question “Have you experienced rape?”, the questionnaire was comprised of four questions referring to other forms of sexual violence or coercive sexual experiences: being forced to have sex with a boyfriend, being forced to have sex with someone you know, being forced to have sex in exchange for goods, and being forced to marry. These four forms of coercive sexual experience are all mentioned as being “sexual violence” in 2006 Congolese legislation (51).

Third, the Adolescent Complex Emergency Daily Stressors Scale (ACEDSS) (50) inquired about a range of different daily and social stressors (stigmatization) and whether or not they occurred during the past month (yes/no). This included 14 daily stressors (e.g., lack of food or medical care), and 14 stigmatization items (perceived discrimination and social exclusion in the familial and community context) (e.g., being treated as if you were different, being isolated by the nuclear family, being treated badly by family members). These stigmatization items were initially derived from the Everyday Discrimination Scale (52), and adapted to this particular cultural context following the procedure of Mels and colleagues (50).

Fourth, perceived social support was measured using the Multidimensional Scale of Perceived Social Support (MSPSS) (53), a brief self-report measure of subjective assessment of social support. This measure was adapted to the cultural context through the cultural adaptation procedure of Mels and colleagues (50). The scale comprises of 12 items that are scored on a five-point Likert scale, ranging from 1 (not at all) to 5 (a lot), and accompanied by a visual probe. The MSPSS measures the perceived adequacy of support of family, friends, and significant others through three subscales, offering the mean scores of items belonging to the subscale. In the interest of answering the research questions, we only retained the family subscale in further analyses. Cronbach alpha of the MSPSS was measured and proved adequate for all three subscales: friends (.70), family (.77), and significant other (.76).

Fifth, coping strategies were measured using the Kidcope (54), which was previously culturally validated for use with eastern Congolese adolescents by Mels et al. (50). This brief instrument uses 11 items to inquire about the use of 11 different coping strategies (e.g., distraction, social withdrawal, wishful thinking, problem solving, emotional regulation, and social support), by asking respondents to indicate on a four-point Likert scale [from 1 (not at all) to 4 (almost all the time)], how frequently they have applied them during the past month. Subscale and total scores were calculated, as was the two-factor structure proposed by Cheng and Chan (55) of escape-oriented coping (sum of subscales: distraction, social withdrawal, self-criticism, blaming others, wishful thinking, resignation, emotional outburst) and control-oriented coping (subscales: problem solving, cognitive restructuring, social support, relaxation). This categorization has been previously used in the eastern Congolese context when studying coping in war-affected adolescents (50) and matches the engagement/disengagement (28) and positive/negative (56) dimension of coping used in coping studies. Cronbach's alpha for the avoidant/disengagement subscale was (.74), yet weaker for the control-oriented/engagement scale (.65). Therefore, we focused on the avoidance/disengagement scale in further analyses.

Sixth, symptoms of posttraumatic stress were measured with the culturally adapted Congolese (French) version (50) of the Impact of Event Scale-Revised (IES-R) (57), a diagnostic self-administered questionnaire comprised of 22 questions to be answered on a Likert scale [from 0 (never) to 5 (extremely)], accompanied by a visual probe. Items can be grouped into three subscales (symptoms of intrusion, avoidance, and hyperarousal). Cronbach's alphas in this study were between.77 and.83.

Seventh, the culturally adapted Congolese (French) version (50) of the Hopkins Symptom Checklist-37 for Adolescents (HSCL-37A) (57) measured symptoms of anxiety (12 items), depression (13 items), and externalising problems (12 items). All items had to be answered on a four-point Likert scale [from 1 (not/never) to 4 (always)], accompanied by a visual probe. Cronbach's alphas in this study were between.60 and.85. The externalizing scale with a low Cronbach alpha of.60 was omitted from further analyses.

### Statistical Analysis

Chi square and ANOVA analysis were carried out to explore differences in sociodemographic variables and types of sexual violence for categorical and continuous variables respectively. Differences between mental health outcomes (HSCL-37A and IES-R) for different types of sexual violence were investigated through ANOVA analysis. Odds ratios were considered to measure differences between groups of type of sexual violence concerning potentially traumatic war-related events. Pearson correlations between covariates were calculated for each group based on the type of sexual violence experienced.

Hierarchical multiple regression analysis was conducted respectively, as dependent variables, with: HSCL-37A subscales of depression and anxiety; the IES-R subscales intrusion, hyperarousal, and avoidance; and the total IES-R posttraumatic stress score. The number of daily stressors (ACEDSS) and war-related traumatic events (ACEES) were entered at stage one of the regression analysis in order to control for these potential risk factors. Stigmatization (number of social stressors; ACEDSS) was entered at stage two, avoidant/disengagement coping (Kidcope) at stage three and family support (MSPSS) at stage four. Prior to model fitting, variables were standardized. To avoid complexity, models were fitted to three subsets of the data based on the experiences of sexual violence (no sexual violence experienced, sexual violence labeled as “rape,” sexual violence labeled as “nonconsensual” sexual experiences), resulting in three times six hierarchical regression models.

Finally, several ANCOVA models were defined to explore the main effects and possible interaction effects of avoidant/disengagement coping and family support with daily stressors, war-related traumatic exposure and stigmatization on the different mental health outcomes. Again, covariates were standardized prior to the analyses.

To control type-I errors, alpha was set at.01.

## Results

### Socio-Demographic Variables and Stressful Experiences

38.2% (n = 499) of adolescent girls who participated in this study reported being a victim of sexual violence. The remaining 61.8% (n = 806) did not report any form of sexual violence. Socio-demographic characteristics of the three groups of participants (i.e., those who did not experience sexual violence, those who did label the sexual violence as “rape,” and those who reported experiences of sexual violence but did not label it as “rape”) and the stressful events they experienced (i.e., war-related traumatic events, daily stressors, and social stressors/stigmatization) are reported in **Table 1**.

Analysis shows that girls who report rape and girls who report NCSE also report more potentially traumatic war-related events than girls who never experienced sexual violence. **Table 2** shows differences between groups on the experiences of potentially traumatic war-related events. Some potentially traumatic war-related events are reported considerably more by rape victims than by NCSE or girls who never experienced sexual violence such as having been in imprisoned, having been kidnapped and enrolled by an armed group, having been forced to kill, injure or rape someone or seeing someone being raped.

**Table 2:** Potentially traumatic war-related events.

	Rape (n=183)	Nonconsensual sex (n=315)	*χ² (df=2)*
Have been separated from family	1.85	2.18	29.38**
Have witnessed violent acts against family members or friends	2.17	1.72	16.29**
Had family members or friends violently killed during the war	1.92	1.19	15.76**
Experienced pillage or setting your house on fire	2.56	1.14	30.15**
Experienced gunfire attacks	2.65	1.74	41.06**
Have seen somebody being killed	2.34	2.20	45.92**
Have seen dead bodies or mutilated bodies	3.21	1.67	53.79**
Have been injured during the war	7.56	2.75	79.69**
Have been in prison	48.18	9.11	183.22**
Have been enrolled in an armed group	50.76	18.22	95.73**
Have been kidnapped by an armed group	19.17	2.99	165.01**
Have been forced to kill, injure or rape someone themselves	8.45	1.42	85.67**
Have seen someone being raped	5.08	2.56	72.79**
*Total traumatic exposure (regression coefficients as obtained from ANOVA)*	2.43	1.01	96,59**

**p < .001.

### Coping Strategies, Family Support and Mental Health

Levels of mental health issues (symptoms of anxiety, depression, and posttraumatic stress) (HSCL-37A and IES-R), avoidant/disengagement coping (Kidcope), and family support (MSPSS) for the three groups are reported in **Table 3**.

**Table 3:** Mental health, family support, and coping.

	Total group (n=1,304)	NSV (n=806)	Rape (n=183)	NCS (n=315)	F
IES-R					
Intrusion	1.82 (.69)	1.71 (.63)	1.83 (.69)	2.09 (.79)	35.94**
Avoidance	1.92 (.72)	1.80 (.70)	2.06 (.68)	2.14 (.76)	29.09**
Hyperarousal	1.87 (.74)	1.71 (.68)	2.08 (.67)	2.15 (.83)	51.19**
Total PTSD score	1.87 (.65)	1.74 (.61)	1.98 (.57)	2.12 (.71)	17.55**
HSCL-37A					
Depression	1.68 (.35)	1.61 (.33)	1.76 (.36)	1.77 (.37)	29.20**
Anxiety	1.76 (.37)	1.71 (.37)	1.79 (.37)	1.85 (.38)	17.18**
Family support (MSPSS)	3.02 (1.01)	3.10 (1.05)	2.85 (.81)	2.96 (1.00)	5.29*
Avoidant/adjustment coping (Kidcope)	1.80 (.52)	1.70 (.48)	2.03 (.54)	1.95 (.53)	50.22**

Mean(SD); *p < .01, **p < .001; NSV, participants who reported no sexual violence; Rape, participants who reported experiences of rape; NCS, participants who reported nonconsensual sexual experiences, but did not label these as “rape”; IES-R, Impact of Events Scale-Revised; PTSD, posttraumatic stress disorder; HSCL-37A, Hopkins Symptom Checklist-37 for Adolescents; MSPSS, Multidimensional Scale of Perceived Social Support.

Pearson's correlations suggested that avoidant/disengagement coping was also correlated to war-related traumatic exposure, daily stressors and stigmatization in both girls who did not report any sexual violence and girls who reported rape (**Table 4**). For all three groups, girls who report sexual violence, girls who report NCSE and those who not report any sexual violence, there is no significant correlation for war-related traumatic exposure, daily stressors, and stigmatization. Strongest correlations were found between stigmatization and daily stressors.

**Table 4:** Pearson correlations between several independent and dependent variables.

		Daily Stressors	War-related trauma	Stigmatization	Family support
War-related traumatic exposure (ACEES)	Total group	.358**			
	NSV	.310**			
	Rape	.186			
	NCSE	.235			
Stigmatization (ACEDSS)	Total group	.520**	.317**		
	NSV	.394**	.223**		
	Rape	.554**	.044		
	NCSE	.410**	.245**		
Family support (MSPSS)	Total group	−.064	−.007	−.071*	
	NSV	−.009	.029	−.058	
	Rape	−.059	−.084	−.162	
	NCSE	−.076	.089	.110	
Avoidant/disengagement coping (Kidcope)	Total group	.249**	.249**	.258**	.125**
	NSV	.209**	.210**	.170**	.177**
	Rape	.304**	.264**	.394**	.098
	NCSE	.033	.062	−.007	.131

*p < .01, **p < .001; NSV, participants who reported no sexual violence; Rape, participants who reported experiences of rape; Nonconsensual sex, participants who reported nonconsensual sexual experiences, but did not label these as “rape”; ACEES, the Adolescent Complex Emergency Exposure Scale; ACEDSS, Adolescent Complex Emergency Daily Stressors Scale; MSPSS, Multidimensional Scale of Perceived Social Support.

### The Impact of Coping and Family Support on Mental Health

Multiple regression analyses (**Table 5**) demonstrated a positive impact of stigmatization on girls' mental health, specifically for those girls reporting NCSEs and those girls who did not report any experiences of sexual violence. This means that the more girls are stigmatized the more mental health problems they report. The results suggest that for girls who report NCSE, the more stigma they experience the more PTSD symptoms they report. For girls who do not report any sexual violence, we found that the more stigma they report, the more depression and anxiety they experience. No main effect of stigma was found in rape victims.

**Table 5:** Multiple hierarchical regression analyses.

	IES-R												HSCL-37A					
	Intrusion			Avoidance			Hyperarousal			PTSD Total			Depression			Anxiety		
STEP 1	NSV	RAPE	NCS	NSV	RAPE	NCS	NSV	RAPE	NCS	NSV	RAPE	NCS	NSV	RAPE	NCS	NSV	RAPE	NCS
War trauma	.35**	.23**	.44**	.29**	.18**	.25**	.34**	.25**	.34**	.36**	.24**	.38**	.23**	.19*	.22**	.21**	.20**	.23**
Daily stressors	.26**	.00	.32**	.26**	.06	.30**	.29**	.20**	.35**	.30**	.08	.35**	.29**	.06	.31**	.27**	.04	.33**
R²	.22**	.09*	.27**	.15**	.08*	.17**	.23**	.21**	.23**	.23**	.14**	.27**	.18**	.07*	.15**	.12**	.07*	.19**
STEP 2																		
War trauma	.34**	.22**	.40**	.27**	.18*	.24**	.33**	.26**	.30**	.34**	.24**	.35**	.20**	.20**	.19**	.18**	.20**	.22**
Daily stressors	.23**	.06	.21**	.21**	.06	.27**	.26**	.13	.27**	.26**	.09	.27**	.21**	−.05	.24**	.19**	−.00	.30**
Stigma	.10	−.09	.28**	.17**	−.01	.08	.10	.10	.23**	.14*	−.02	.22**	.30**	.17	.19*	.26**	.06	.08
Delta R²	.01	.01	.05**	.01**	.00	.00	.01	.01	.03**	.01*	.00	.03**	.05**	.03	.03*	.03**	.00	.00
STEP 3																		
War trauma	.30**	.22**	.39**	.23**	.15*	.22**	.28**	.19**	.29**	.30**	.21**	.33**	.17**	.16*	.18*	.16**	.16*	.21**
Daily stressors	.20**	.06	.20*	.18**	.07	.26**	.22**	.10	.26**	.22**	.08	.26**	.19**	−.06	.23**	.18**	−.02	.30**
Stigma	.07	−.10	.29**	.14*	−.04	.09	.07	.00	.23**	.11*	−.06	.23**	.29**	.10	.19*	.25**	−.01	.08
Avoidant coping	.22**	.01	.17*	.24**	.23*	.33**	.27**	.35**	.18**	.27**	.16	.26**	.16**	.23*	.17*	.13**	.23*	.15*
Delta R²	.05**	.00	.02*	.05**	.04*	.11**	.07**	.12**	.03**	.07**	.03	.06**	.02**	.04*	.03*	.01**	.05*	.02*
STEP 4																		
War trauma	.30**	.24**	.37**	.23**	.16*	.22**	.28**	.20**	.29**	.30**	.22**	.33**	.17**	.15	.18**	.16**	.15	.21**
Daily stressors	.20**	.05	.24**	.18**	.07	.27**	.22**	.10	.26**	.22**	.07	.28**	.18**	−.06	.23**	.18**	−.02	.29**
Stigma	.07	−.06	.26**	.15*	−.03	.09	.07	.02	.23**	.11*	−.04	.21**	.28**	.07	.20*	.25**	−.02	.09
Avoidant coping	.23**	−.03	.15*	.23**	.21	.33**	.27**	.33**	.18**	.27**	.13	.25**	.17**	.26*	.17*	.13**	.24*	.15*
Family support	−.03	.23	.19**	.02	.08	.04	.00	.09	−.00	.00	.16*	.09	−.06*	−.14	−.01	−.01	−.08	−.03
Delta R²	.00	.03	.03**	.00	.01	.00	.00	.01	.00	.00	.02*	.01	.01*	.01	.00	.00	.00	.00

*p <. 01, **p <. 001; NSV, participants who reported no sexual violence; Rape, participant who reported experiences of rape; Nonconsensual sex, participants who reported nonconsensual sexual experiences, but did not label these as “rape.”

The change in R² from model 2 to 3 showed that avoidant/disengagement coping explained a substantial amount of variance to the previous models, in particular for girls who had not experienced sexual violence and girls who reported NCSEs. For girls who reported rape, avoidant/disengagement coping only led to more symptoms of hyperarousal (IES-R),

For all participants there is a significant positive effect of avoidant/disengagement coping on symptoms of depression and anxiety (HSCL-37A). This means the more avoidant/disengagement coping they apply the more depression and anxiety (HSCL-37A) symptoms they report.

There is a positive effect of avoidant/disengagement coping for girls who do not report sexual violence and to a smaller extent for girls who report NCSE. Avoidant/disengagement coping has a strong positive effect on avoidance symptoms (IES-R) for all three groups. In addition, a strong positive effect of avoidance coping on hyperarousal symptoms (IES-R) for girls who report rape.

The change in R² from model 3 to 4 showed that family support explained some variance compared to the previous models. Family support shows a strong positive effect on intrusion symptoms for girls who report NCSE. A strong positive effect of family support is also found for PTSD symptoms in girls who report rape. This means rape victims who report more family support also report more PTSD symptoms. In addition, we found a small negative effect of family support on depression for girls who do not report any sexual violence.

**Figure 1:**
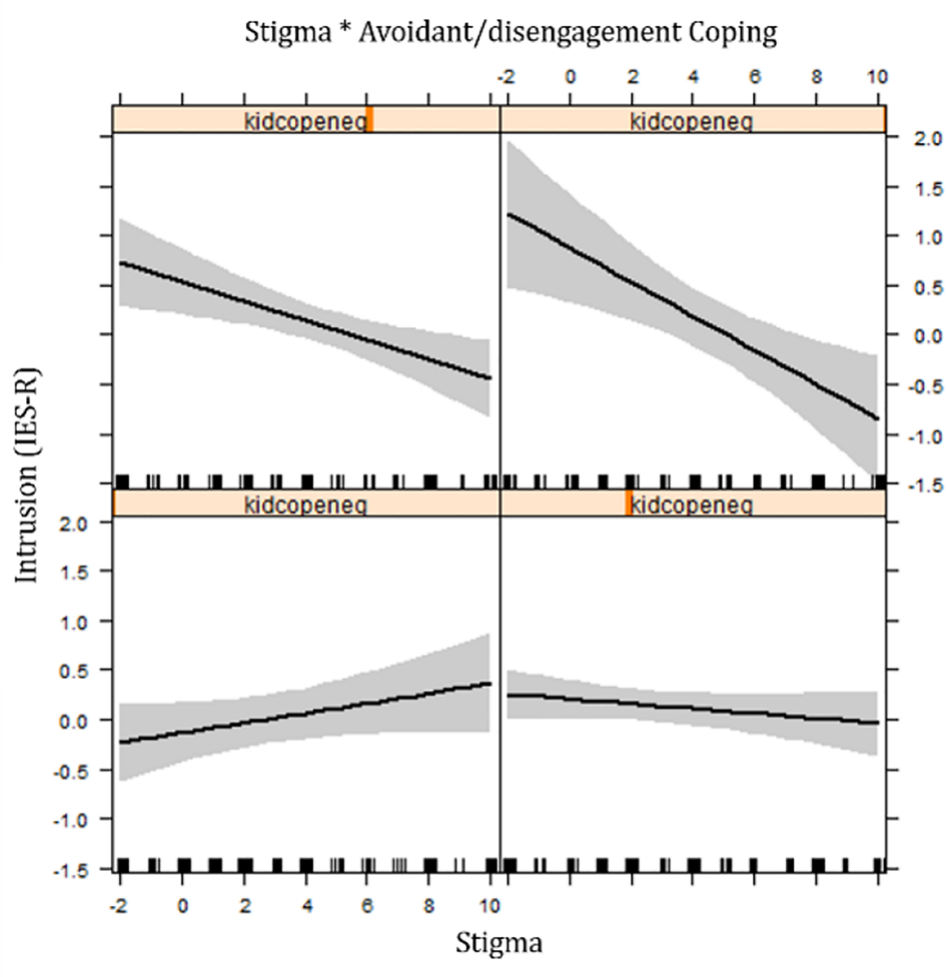
Effect plot showing interaction between avoidant/disengagement coping and stigma on intrusive symptoms for rape victims.

Further exploration of the impact of avoidant/disengagement coping with ANCOVA-analyses showed that there were interaction effects between avoidant/disengagement coping and stigmatization in victims of sexual violence (both girls who report as girls who report NCSE) for symptoms of posttraumatic stress and anxiety (**Table 6**). In lower levels of avoidant coping, the relation between stigmatization and psychological symptoms is slightly positive; when reported avoidant coping is high, the relationship between stigmatization and psychological symptoms is strongly negative (**Figure 1**).

**Table 6:** ANCOVA analyses exploring the impact of avoidant/disengagement coping on mental health.

	IES-R												HSCL-37A					
	Intrusion			Avoidance			Hyperarousal			PTSD total			Depression			Anxiety		
	NSV	RAPE	NCS	NSV	RAPE	NCS	NSV	RAPE	NCS	NSV	RAPE	NCS	NSV	RAPE	NCS	NSV	RAPE	NCS
Intercept	0.01	−0.08	0.15*	0.18	0.06	0.09	−0.02	−0.08	0.16*	0.00	−0.02	.14*	0.03	−0.06	0.08	0.05	−0.10	0.09
Stigma	0.09	−0.08	0.27**	0.16**	−0.05	0.09	0.09	−0.01	0.21**	0.12*	−0.07	.21**	0.29**	0.11	0.25**	0.25**	0.00	0.09
War-related trauma	0.29**	0.24**	0.37**	0.23**	0.14	0.23**	0.28**	0.19**	0.27**	0.29**	0.22**	.33**	0.17**	0.20*	0.15*	0.15**	0.18*	0.23**
Daily stressors	0.22**	0.12	0.15	0.18**	0.07	0.23**	0.23**	0.10	0.23**	0.24**	0.10	.22**	0.18**	−0.03	0.18*	0.18**	0.03	0.26**
Avoidant coping	0.28**	0.35*	0.22**	0.28**	0.10	0.32**	0.31**	0.24	0.25**	0.32**	0.27	.29**	0.17**	0.42*	0.09	0.12*	0.49**	0.09
Stigma * avoidant coping	0.07	−0.27*	−0.15*	0.02	0.01	0.00	0.04	−0.06	−0.18*	0.05	−0.17	−.10	−0.02	−0.00	0.02	−0.10	−0.18	0.08
War-related * trauma avoidant coping	0.06	−0.17*	−0.11	0.11	−0.06	−0.04	0.06	−0.03	−0.09	0.88	−0.11	−.09	0.10	−0.19*	0.12	0.08	−0.16*	−0.01
Daily * stressors avoidant coping	0.07	0.17	0.28**	0.13	0.22	0.12	0.04	0.19	0.22**	0.05	0.21	.22**	−0.03	−0.02	0.12	0.06	0.12	0.10
Overall explained variance (r^2^)	0.28	0.17	0.39	0.23	0.18	0.29	0.31	0.38	0.33	0.32	0.21	0.39	0.23	0.18	0.24	0.17	0.16	0.24

*p < .01, **p <. 001; NSV, participants who reported no sexual violence; Rape, participants who reported experiences of rape; NCS, participants who reported nonconsensual sexual experiences, but did not label these as “rape”; IES-R, Impact of Events Scale-Revised; PTSD, posttraumatic stress disorder; HSCL-37A, Hopkins Symptom Checklist-37 for Adolescents.

In the effect plots, the lower plots are associated with a higher number of avoidant/disengagement coping while the higher plots suggest a higher level of avoidant/disengagement coping. The interaction effects found between avoidant/disengagement coping and daily stressors in victims of sexual violence (rape and NCSE) were: when low avoidance coping was reported no association was found between daily stressors and posttraumatic stress symptoms (IES-R); while in high levels of avoidant coping a positive association was reported between daily stressors and symptoms. In contrast, interaction effects of avoidant/disengagement coping with war-related traumatic exposure for victims of rape on symptoms of intrusion (IES-R), depression, and anxiety (HSCL-37A) were: when low levels of avoidant coping were noticed, there was a strong positive relation between war-related trauma and symptoms; while a reverse relationship was observed in high levels of avoidance coping.

Overall, there seemed to be a limited impact of family support on participants' mental health, as also indicated by the variability accounted for between the third and the fourth models (**Table 7**). Higher levels of family support were significantly associated with symptoms of intrusions in girls who reported experiences of sexual violence.

**Table 7:** ANCOVA analyses exploring the impact of family support.

	IES-R									HSCL-37A								
	Intrusion			Avoidance			Hyper-arousal			PTSD total			Depression			Anxiety		
	NSV	RAPE	NCS	NSV	RAPE	NCS	NSV	RAPE	NCS	NSV	RAPE	NCS	NSV	RAPE	NCS	NSV	RAPE	NCS
Intercept	0.01	−0.10	0.20**	0.01	0.03	0.18	−0.04	−0.11	0.17*	−0.00	−0.06	0.20**	0.02	−0.07	0.13	0.05	−0.13	0.12
Stigma	0.10	−0.05	0.23**	0.18**	0.00	0.05	0.11*	0.14	0.19*	0.14**	0.01	0.18*	0.30	0.17	0.19*	0.27**	0.07	0.07
War-related trauma	0.34**	0.21**	0.36**	0.27**	0.07	0.22**	0.32**	0.25**	0.28**	0.34**	0.23**	0.32**	0.20	0.17*	0.20**	0.18**	0.17**	0.21
Daily stressors	0.23**	0.09	0.24**	0.22**	0.02	0.28**	0.26**	0.14	0.26**	0.26**	0.11	0.29**	0.21	−0.02	0.24**	0.20**	0.02	0.30
Family support	0.03	0.18	0.18**	0.08	0.17	0.07	0.08	0.14	−0.00	0.07	0.18	0.10	−0.03	−0.05	0.02	0.02	0.02	0.00
Stigma* family support	0.02	−0.00	0.06	0.04	0.02	0.04	0.04	0.03	0.17*	0.36	−0.01	0.08	0.01	0.08	−0.10	0.03	0.03	−0.02
War-related trauma* family support	0.03	−0.06	0.18**	0.02	−0.02	0.17*	0.02	−0.06	0.28**	0.28	−0.06	0.22**	−0.02	−0.14	−0.02	−0.00	−0.11	0.14
Daily stressors* family support	0.01	0.23*	0.05	0.00	0.03	0.05	0.02	0.15	−0.02	0.01	0.18	0.04	−0.00	0.06	0.02	−0.00	0.07	−0.30
Overall explained variance (r^2^)	0.22	0.17	0.39	0.17	0.11	0.21	0.24	0.28	0.37	0.25	0.20	0.37	0.21	0.13	0.19	0.15	0.10	0.20

*p < .01, **p < .001; NSV, participants who reported no sexual violence; Rape, participants who reported experiences of rape; NCS, participants who reported nonconsensual sexual experiences, but did not label these as “rape”; IES-R, Impact of Events Scale-Revised; PTSD, posttraumatic stress disorder; HSCL-37A, Hopkins Symptom Checklist-37 for Adolescents.

**Figure 2:**
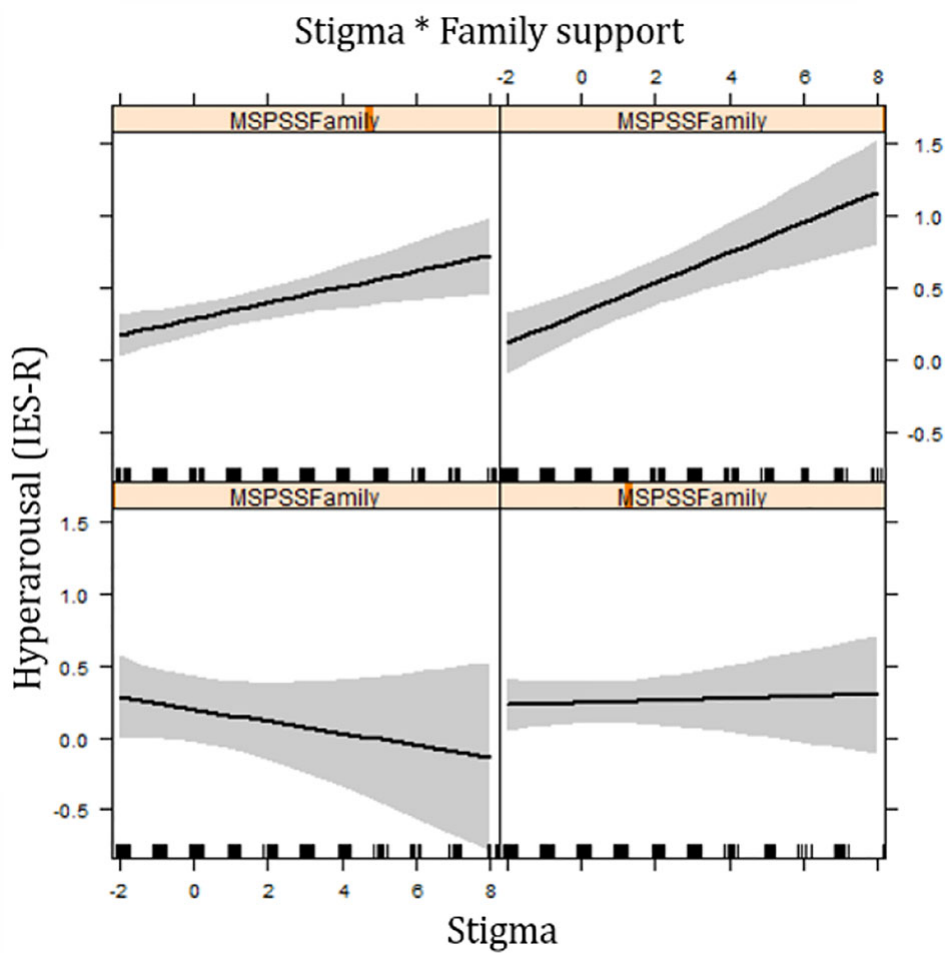
Effect plot showing interaction between family support and stigma on hyperarousal symptoms for girls reporting NCSE.

Further analyses indicated that for girls who reported NCSEs there was a strong interaction effect between family support and stigmatization on hyperarousal symptoms (IES-R): for victims who reported low levels of family support, stigmatization was not significantly associated with higher hyperarousal symptoms; while for girls with estimated high levels of family support, an increase in stigmatization was strongly associated with an increase in hyperarousal symptoms (**Figure 2**).

For girls who reported rape, an interaction effect was found in intrusion symptoms (IES-R) between family support and daily stressors: there was no association between family support and intrusion symptoms when little daily stressors are mentioned; however, when reporting higher levels of daily stressors, an increase in family support was associated with higher levels of intrusion.

In the group of girls who reported NCSEs, analyses showed interaction effects between family support and war-related traumatic exposure on all posttraumatic stress symptoms: when low levels of war-related trauma was reported, there was no association between family support and posttraumatic stress; while in high levels of war-related traumatic exposure, an increase of family support was strongly associated with posttraumatic stress.

## Discussion

Over one-third of adolescent girls in this study reported having experienced sexual violence. While sexual violence clearly impacts adolescent girls' mental health, a considerable variation in trauma symptoms can still be observed. This study investigated the potentially protective role of avoidant/disengagement coping and family support for adolescent girls in Eastern Congo who reported they were victims of sexual violence (either “rape” or “NCSEs”), in comparison with girls who did not report any sexual violence. While this study underscores the role of both avoidant/disengagement coping and family support in girls' mental well-being after sexual violence, it also revealed important interactions with other factors, like stigmatization.

First, for girls who did not report any experiences of sexual violence *avoidant/disengagement coping* has a direct negative effect on all psychological outcomes, which underscores the well-documented negative effect of avoidant/disengagement coping on psychological distress (29, 31–33).

However, a more nuanced picture about the influence of avoidant coping was found in girls who reported experiences of sexual violence. An interaction effect between avoidant/disengagement coping and stigmatization on different mental health outcomes (posttraumatic stress symptoms and anxiety) was found. This means that when the girls experienced little stigma, avoidant/disengagement coping seemed to impact mental health negatively. Although, when girls reported high levels of stigma, avoidant/disengagement coping seemed to serve as a protective factor, as it is associated with a lower level of psychological symptoms. One possible explanation here is that stigmatization accounts for a continuous revictimization (58–60), creating a situation in which avoidant/disengagement coping is seemingly an adaptive way to deal with these overwhelming emotional responses (38, 61, 62). A qualitative study in Eastern Congo (63) corroborates these findings, describing how adolescent girls identified the advice to cope with sexual violence and its social consequences in an avoidant way as the most helpful strategy to overcome their psychological difficulties. These findings also add to a more nuanced perspective on avoidant coping that goes beyond a traditional divide of avoidant coping being either harmful or helpful (64).

In contrast with the impact of a high number of experiences of stigmatization we found that in girls who experience high levels of daily stressors, avoidant/disengagement coping strategies were associated with a strong increase in posttraumatic stress symptoms. So, in the case the victim finds herself in a situation of overwhelming material and situational daily stressors, it adds an additional burden and thoroughly affects their mental health. At the same time, it might not necessarily retraumatize the victim or make them relive the trauma creating a situation of prolonged and recurrent traumatization in which avoidant coping might be adaptive and lead to less posttraumatic stress symptoms, as is the case in stigmatization. Furthermore, we hypothesize that avoidant coping might not be very helpful when confronted with high levels of daily stressors, as they pervasively influence their primary needs.

Second, the study showed how girl victims of sexual violence experienced less *family support* than peers who had not experienced sexual violence. In contrast with other studies, we found no main protective role of family support for the mental well-being of girls who experienced sexual violence. These findings thus support studies that point to the complex role families play in supporting victims of sexual violence in (post) conflict settings (65). One hypothesis here could be that the social support questionnaire (MSPSS) did not fully assess all of the socio-cultural meanings of social support (66). Moreover, the MSPSS mainly included emotional support, while recent qualitative studies revealed that adolescent victims of sexual violence in eastern Congo defined social support from family members through a combination of instrumental and emotional supports (23, 63). It is possible that the situational demands, reflected through the numerous daily and social stressors, require a social support that is more instrumental than emotional. Potentially the large presence and activities of humanitarian and development organisations across the region (44) could contribute to a conceptualisation of support and needs in an instrumental way. A second hypothesis is linked to other findings that demonstrated how avoidant/disengagement coping might be an adequate response when dealing with both sexual violence and high levels of stigma or traumatic exposure, a coping strategy that might not be compatible with emotional family support. In addition, sharing pain and difficulties might make the victims feel the pain of their adversity more intensely, and, consequently, be associated with more mental health problems (67).

We also found interaction effects that revealed a more complex role of family support with symptoms of posttraumatic stress, in particular for girls who reported NCSEs: when girls report high levels of support from their family, there was a positive association between stressors (daily stressors, stigma and war-related trauma) and symptoms of PTSD, while this was not the case when low levels of family support were indicated. Interestingly, these interaction effects were not found in girls reporting rape.

One possible explanation for both the main as interaction effects of family support could be that these girls reported higher levels of stigma, not only from the larger community but also from family members. It might be the case that confounded stigmatization or rejection by family members might render family support less helpful for these victims of sexual violence. A recent study with victims of sexual violence in Eastern Congo also pointed to the positive association of emotional support seeking and stigma and symptoms of depression and PTSD (68). Punamäki (69) found that inconsistent social support from parents was related to higher levels of posttraumatic stress disorder, compared to children who perceived overall loving support from both parents. Moreover, sexual violence not only impacts the victim, but also her close social environment (23). In a context where there is still a large stigma attached to sexual violence, disclosure of sexual violence could greatly affect family members' well-being and social position, impeding them from providing social support to the victim (70). Hobfoll and London (71) also proposed the notion of the “pressure cooker effect,” referring to the way, especially in times of armed conflict, social support might backfire. Here, Hobfoll and London (71) point to the way in which social relationships are put under pressure in times of war, as conversations are inundated by recurring rumors, impending doom and needed comfort referring to war, while close intimate social support providers are also struggling with the same problems and, therefore, unable to provide adequate support. Furthermore, providing social support may confront family and community members with their own sense of guilt and shame for failing to prevent what happened to their own daughter or neighbour (67).

### Limitations

It is also important to consider limitations to this study. First, the socio-cultural context in which coping and social support occur may influence the strategies utilized, the extent to which they are (mal)adaptive, and their specific cultural understanding. The questionnaires used, although culturally validated, may not have captured all socio-cultural meanings and interpretations of these protective factors. We recommend future studies that also include further qualitative explorations of the cultural and contextual representation of concepts as social support and coping.

Second, the post-conflict contextual realities directed us toward a cross-sectional study design. A longitudinal study would have provided information on participants' previous psychological well-being and on the long-term influence of adherence to particular coping strategies or reliance on family support.

Third, while sexual violence was assessed in different forms (rape, NCSEs), the reported figures are most likely an underestimation of the true extent of sexual violence experienced by the participants. Research has shown that sexual violence is often underreported in the war-ridden region as victims fear accusation and stigma (72–74). On the other hand, there is a possibility that for some respondents their reporting of sexual violence was informed by an expectation of material compensation. However, throughout the study it was repeatedly stressed that no material compensation was connected to reporting.

Practical reasons and logistics made that only school-going girls were included in the study, this might diminish the study generalizability to out-of-school adolescents.

### Implications

An ecological approach to mental health sequalae of sexual violence in adolescent victims in a war-ridden setting like eastern Congo is scarce. Building on the findings of this study, we seek to formulate important implications for further research and intervention.

First, our study shows that the effect of avoidance/disengagement coping on mental health outcomes of victims of sexual violence is not linear, nor fixed. Second, the flexible adaptation of coping strategies by adolescent girl victims of sexual violence speaks to the adoption of flexible approaches in providing psychological care. As situational demands and factors, such as daily stressors and stigmatization, are strongly associated with the use of coping strategies, psychological support needs to consider these extra burdens on victims' mental health. Further research on other coping strategies, beyond avoidance/disengagement coping, would be crucial to further the comprehension of coping strategies that could be addressed in psychological care initiatives.

Third, while these results suggest that family support might not serve as a protective factor for mental health in most adolescent girls, we plead not to discard family support in the ecological investigation of risk and protective factors on mental health outcomes of sexual violence. We thus plea for more interventions addressing the psychosocial well-being of family and community members in order to support them to create an adapted supportive environment for their victimised family and community members. Above all, we urge for further investigation of the nature of “adaptive” or “helpful” family support in the complex process of recovery from sexual violence, hereby taking into account the specific socio-cultural and contextual ideas. This further investigation of the socio-cultural understanding of the mental health impact of sexual violence, as well as the role of social and family support and other protective resources, should inform local support initiatives for victims of sexual violence. Informal care structures such as religious support groups, traditional support mechanisms and peer support groups seem to play an important role in the well-being of victims of sexual violence (63). These informal care structures and mechanisms merit further scientific explorations to inform local support initiatives.

Fourth, we suggest a holistic approach to healing when taking into account family support and coping strategies. Our research shows that victims of sexual violence face an array of difficulties, from daily stressors to stigmatization. These findings combined with the need for extensive attention to the socio-cultural meaning of social support, direct us to propose a systems approach that considers the individual definition of helpful psychosocial support. Instrumental support might in some cases regarded as more adapted to an individual victim's needs, and form more a perceived priority than strengthening emotional family support. Therefore, we think interventions supporting victims of sexual violence should include attention for instrumental needs while respecting the potential protective role of the family and their support mechanisms.

## Conclusions

Over one-third of adolescent girls who participated in this study reported experiencing sexual violence. The study focused on the impact of avoidant/disengagement coping and family support on the mental health sequelae of the experience of sexual violence in eastern Congolese girls. Analysis showed that that negative coping in this postconflict setting resulted in more negative mental health outcomes, corroborating with the thesis that long-term adherence to avoidant coping increases negative mental health outcomes. In victims of sexual violence, however, the combination of having experienced sexual violence and enduring high levels of stigmatization or having been exposed to more potentially traumatic life-events created a reverse effect, where avoidant/disengagement coping leads to less posttraumatic stress. In addition, this study does not find evidence that family support is a protective factor for mental health outcomes after sexual violence. To the contrary, in girls who report NCSE we found that when high levels of family support were reported, a positive association between stressors (daily stressors, stigma, and war-related trauma) and posttraumatic stress symptoms exists. The study underscores the importance of further scientific investigation into the complex role of coping and family support in the mental health consequences of sexual violence in adolescent victims.

## Author’s Note

The study described in this manuscript is part of a large-scale quantitative study conducted in Eastern Democratic Republic of the Congo (DRCongo) with 1304 school-going adolescent girls. The aim of the larger doctoral study is to further evidence base on the psychological and social consequences of sexual violence toward adolescent victims in the war-affected region of Eastern DRCongo. A first published study by Verelst, De Schryver, Broekaert, and Derluyn (21) focuses on the association of how war-affected adolescent girls label sexual violence (rape or NCSEs) and their mental health. This study documents the impact of different risk factors such as daily stressors and stressful life experiences on the mental health sequalae of sexual violence. A second publication (20) drawing on the large quantitative data set focused on the stigmatization and showed that in this population the mental health impact of sexual violence is largely explained by stigmatization.

This particular study indeed builds on the previous studies but has a distinguished novel focus. While the methodology to the study overlaps with previous studies because the dataset it describes is to some extent the same, the analysis and focus are distinct. This study zooms in onto the role of two risk and protective factors to the mental health impact of sexual violence. Avoidant/disengagement coping is generally perceived as a risk factor and family support generally perceived as a protective factor for mental health outcomes of sexual violence. In none of the previous studies these factors have been addressed. Some of the variables such as stigma, daily stressors and war-related traumatic events are also part of this study to allow for ANCOVA models that explore interaction models, hence the overlap in the methodology section with previous publications. The analysis is different than any of the other studies also shedding light on new results. These results seek to contribute to the specific evidence base on the mental health impact of sexual violence in adolescent victims related to the association with avoidant/disengagement coping and family support.

## Data Availability Statement

The datasets presented in this article are not readily available because the informed consents did not mention sharing of the data beyond the research team, we will unfortunately not be able to share the dataset even upon request.

## Ethics Statement

The studies involving human participants were reviewed and approved by Ethical Committee of the Faculty of Psychology and Educational Sciences, Ghent University. Written informed consent from the participants' legal guardian/next of kin was not required to participate in this study in accordance with the national legislation and the institutional requirements.

## Author Contributions

All authors contributed to the design of the study, the data collection and analysis and the writing up of the article.

## Funding

This study received financial support from Service Peace Building, Ministry of Foreign Affairs, Foreign Trade and Development Cooperation, Belgium.

## Conflict of Interest

The authors declare that the research was conducted in the absence of any commercial or financial relationships that could be construed as a potential conflict of interest.
